# CX_3_CR1 Is Expressed by Human B Lymphocytes and Meditates CX_3_CL1 Driven Chemotaxis of Tonsil Centrocytes

**DOI:** 10.1371/journal.pone.0008485

**Published:** 2009-12-29

**Authors:** Anna Corcione, Elisa Ferretti, Maria Bertolotto, Franco Fais, Lizzia Raffaghello, Andrea Gregorio, Claudya Tenca, Luciano Ottonello, Claudio Gambini, Glaucia Furtado, Sergio Lira, Vito Pistoia

**Affiliations:** 1 Laboratory of Oncology, IRCCS G. Gaslini, Genova, Italy; 2 Laboratory of Phagocyte Physiopathology and Inflammation, Department of Internal Medicine, University of Genoa, Genoa, Italy; 3 Human Anatomy Section, Department of Experimental Medicine, University of Genoa, Genoa, Italy; 4 Human Pathology Section, IRCCS G. Gaslini, Genoa, Italy; 5 Immunobiology Center, Mount Sinai School of Medicine, New York, New York, United States of America; New York University, United States of America

## Abstract

**Background:**

Fractalkine/CX_3_CL1, a surface chemokine, binds to CX_3_CR1 expressed by different lymphocyte subsets. Since CX_3_CL1 has been detected in the germinal centres of secondary lymphoid tissue, in this study we have investigated CX_3_CR1 expression and function in human naïve, germinal centre and memory B cells isolated from tonsil or peripheral blood.

**Methodology/Principal Findings:**

We demonstrate unambiguously that highly purified human B cells from tonsil and peripheral blood expressed CX_3_CR1 at mRNA and protein levels as assessed by quantitative PCR, flow cytometry and competition binding assays. In particular, naïve, germinal centre and memory B cells expressed CX_3_CR1 but only germinal centre B cells were attracted by soluble CX_3_CL1 in a transwell assay. CX_3_CL1 signalling in germinal centre B cells involved PI3K, Erk1/2, p38, and Src phosphorylation, as assessed by Western blot experiments. CX_3_CR1^+^ germinal centre B cells were devoid of centroblasts and enriched for centrocytes that migrated to soluble CX_3_CL1. ELISA assay showed that soluble CX_3_CL1 was secreted constitutively by follicular dendritic cells and T follicular helper cells, two cell populations homing in the germinal centre light zone as centrocytes. At variance with that observed in humans, soluble CX_3_CL1 did not attract spleen B cells from wild type mice. OVA immunized CX_3_CR1^−^/^−^ or CX_3_CL1^−^/^−^ mice showed significantly decreased specific IgG production compared to wild type mice.

**Conclusion/Significance:**

We propose a model whereby human follicular dendritic cells and T follicular helper cells release in the light zone of germinal centre soluble CX_3_CL1 that attracts centrocytes. The functional implications of these results warrant further investigation.

## Introduction

CX_3_C chemokine ligand 1 (CX_3_CL1) is a multidomain molecule consisting of a chemokine domain linked to a transmembrane domain via an extended mucin-rich stalk, and of an intracellular domain [1]. CX_3_CL1, that exists as membrane-anchored and soluble forms, is constitutively expressed in many hematopoietic and non-hematopoietic tissues [2], [3], [4], [5].

CX_3_C chemokine receptor 1 (CX_3_CR1), the exclusive CX_3_CL1 receptor, is a pertuxis toxin (PTX)-sensitive seven-transmembrane G protein-coupled receptor (GPCR) expressed on human NK cells, monocytes, Th1 CD4^+^ cells, CD8^+^ T cells and mast cells [6]. Two types of interactions between CX_3_CL1 and CX_3_CR1 have been reported, one occurring between the membrane anchored forms of CX_3_CL1 and CX_3_CR1, the other one between surface CX_3_CR1^+^ and soluble CX_3_CL1. Membrane-bound CX_3_CL1 induces firm adhesion of leukocytes under static and flow conditions without activating integrins [7], whereas soluble CX_3_CL1 released from the cell surface following proteolytic cleavage [1], [3] induces chemotaxis of CX_3_CR1^+^ cells.

Previous studies failed to detect CX_3_CR1 expression in human B lymphocytes [6], [8], [9]. Since CX_3_CL1 is expressed in secondary lymphoid follicles [2], we reasoned that this chemokine could be involved in the local B cell trafficking and decided to re-investigate with different approaches the expression of CX_3_CR1 in the major B cell subsets from tonsil and peripheral blood. We show that indeed CX_3_CR1 is expressed by human naïve, memory and germinal center (GC) B cells, that are the only subset attracted by soluble CX_3_CL1 in chemotaxis assays.

The GC is the site where antigen activated naïve B cells migrate, proliferate and undergo class switch recombination, antibody gene diversification and affinity maturation [10]. GC have two distinct zones called dark and light zones based upon histological appearance. B cells in the dark zone, called centroblasts, proliferate and somatically hypermutate antibody variable genes, then move to the light zone where they are selected based on the affinity of the B cell receptor for antigen [10]. Recent studies have delineated two alternative models for B cell trafficking in the GC, i) the cyclic re-entry model, whereby B cells that have migrated from the dark to the light zone and have been selected subsequently return to the dark zone for further proliferation and, ii) the intrazonal recirculation model, whereby most B cells stay in dark or light zones and selection in these areas operates independently. This latter model keeps into account the low frequency of GC B cells recirculating from one zone to the other [11], [12], [13], [14], [15].

These mechanisms have been identified in mouse models [11], [12], [14], [15], [16], whereas less is known on human GC B cell trafficking [17], [18]. This study adds a new piece of information on the latter issue.

## Results

### Expression and Function of CX_3_CR1 in Human Tonsil B Cells

We first investigated CX_3_CR1 expression in highly purified human B cells from tonsil and peripheral blood by flow cytometry. Ten independent experiments demonstrated that B cells from both sources expressed CX_3_CR1 (tonsil B cells median 46%, range 35–70; median MRFI 5.2, range 4.0–8.0; blood B cells median 52%, range 36–59; median MRFI 6.0, range 4.0–7.0). In spite of a trend to a higher MRFI in blood *vs* tonsil B cells the difference was not statistically significant.

**Figure 1:**
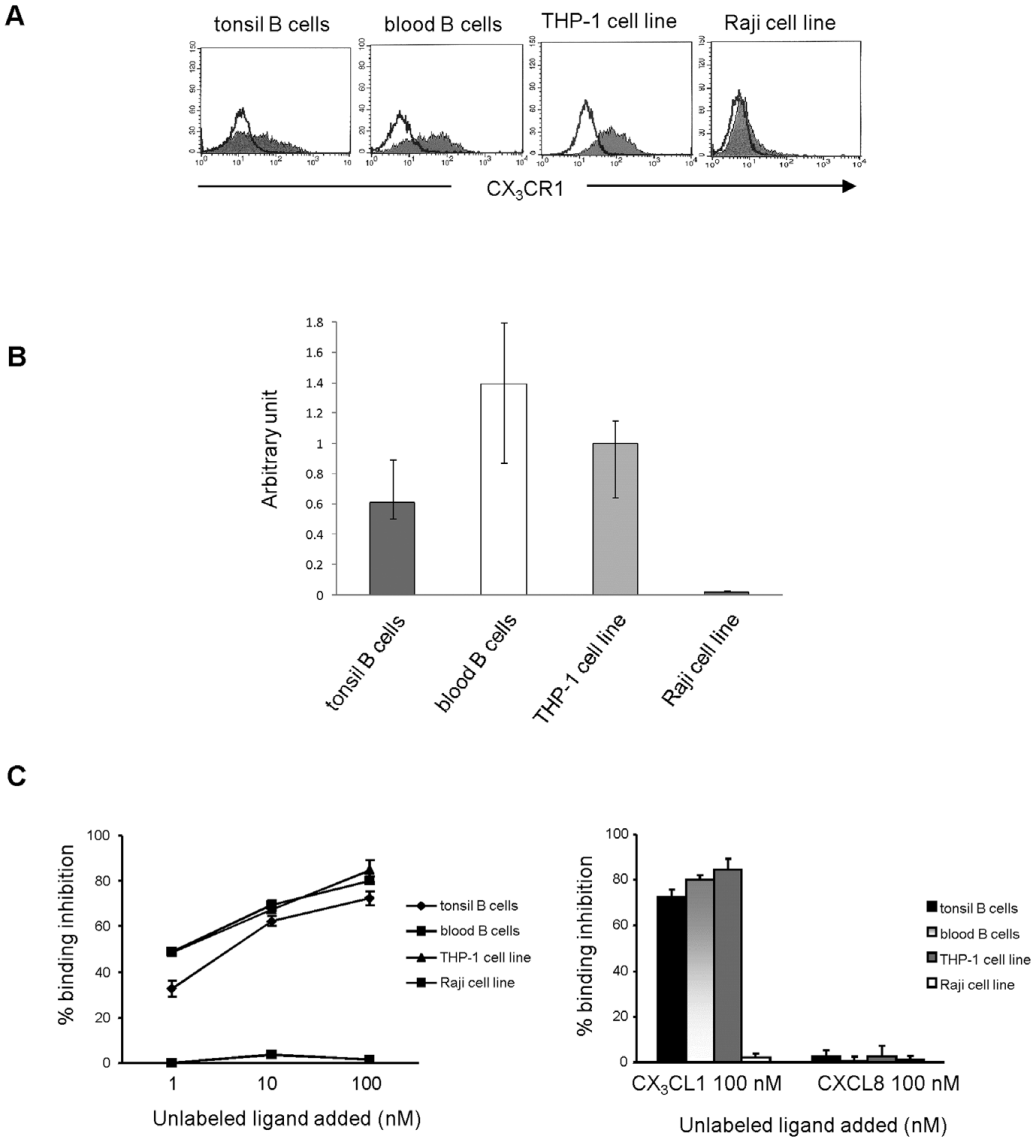
Expression of CX_3_CR1 in tonsil and blood B cells. (**A**) Flow cytometric analysis of CX_3_CR1 expression in tonsil and blood B cells. Representative histograms from each B cell fractions, and THP-1 and Raji cell lines, tested as positive and negative controls, respectively, are shown. (**B**) Quantization of CX_3_CR1 by real time PCR in peripheral blood and tonsil B cells. Data are normalized to the expression of POLR2A. Values are expressed as arbitrary units calculated as fold of CX_3_CR1 expression relative to the THP-1 cell line, arbitrarily set at 1. Raji cell line was tested as negative control. Data are median, minimum and maximum values from two different experiments performed in quadruplicate. (**C**) Displacement experiments of ^125^I-CX_3_CL1 in tonsil and blood B cells. Left panel. Cells were incubated for 2 h at 4°C with 1 nM ^125^I-CX_3_CL1 in the absence or presence of 1, 10 and 100 nM cold CX_3_CL1. Percentage of binding inhibition by unlabeled CX_3_CL1, calculated as ratio between cell-bound cpm in the presence of unlabeled ligand and cell-bound cpm in the absence of unlabeled ligand multiplied by 100, was used as a measure for competition between ^125^I-labeled and unlabelled CX_3_CL1. THP-1 and Raji cell lines were tested as positive and negative controls, respectively. Right panel. The experiments shown in the left panel were repeated using 100 nM cold CXCL8 as negative control.

Representative histograms from tonsil and blood B cell fractions, and control cell lines (THP-1 and Raji, positive and negative controls, respectively) are shown in Fig. 1A.

CX_3_CR1 mRNA expression was next evaluated by real time PCR in tonsil and blood B cells and compared with that in THP-1 cells. As shown in Fig. 1B, CX_3_CR1 mRNA was detected in tonsil B cells and, at higher amount, in blood B cells. Expression of CX_3_CR1 mRNA was never detected in the Raji Burkitt lymphoma cell line (Fig. 1B).

Ligand specificity of CX_3_CL1 binding to CX_3_CR1 was demonstrated by competition binding experiments. Displacement curves show that unlabeled CX_3_CL1 competed for binding of ^125^I-CX_3_CL1 to tonsil and circulating B cells, as well as to THP-1 cell line (positive control), in a dose-dependent manner (Fig. 1C, left panel), showing up to 70% inhibition with 100 nM unlabeled CX_3_CL1. On the contrary, no competition for ^125^I-CX_3_CL1 binding was observed in Raji cell line used as negative control. Finally, 100 nM unlabeled CXCL8, tested as negative control, did not compete for binding of ^125^I-CX_3_CL1 to tonsil and circulating B cells, as well as to THP-1 and Raji cell lines (Fig. 1C right panel).

Taken together, the above experiments demonstrated that B cells expressed unambiguously CX_3_CR1 mRNA and protein.

**Figure 2:**
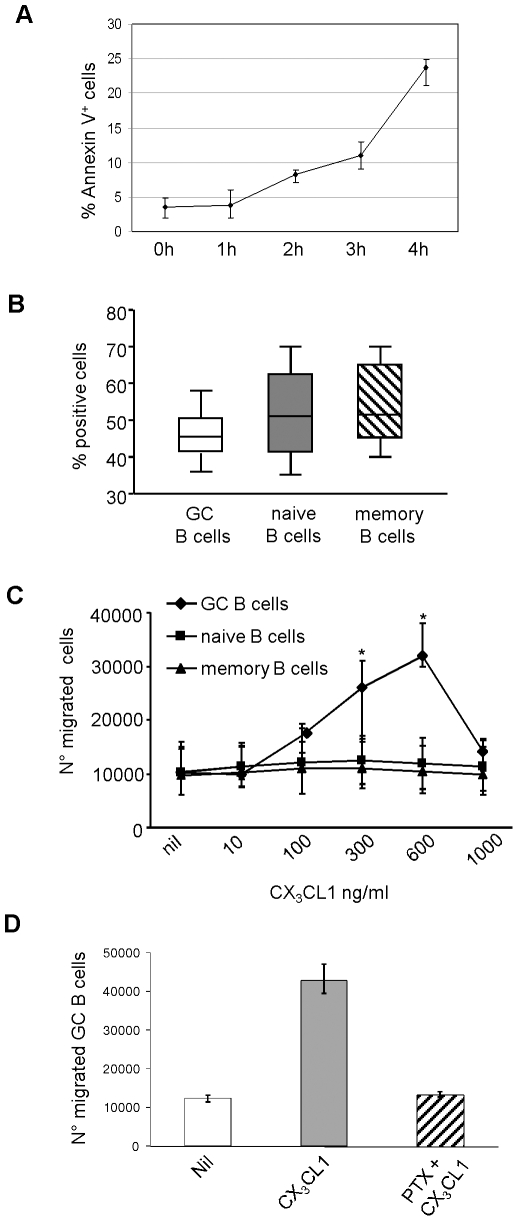
Expression and chemotactic activity of CX_3_CR1 in tonsil B cell subsets. (**A**) Apoptosis evaluation in purified tonsil GC B cells. The proportion of early apoptotic GC B cells was detected by Annexin V staining at time 0 and after 1, 2, 3, and 4h culture. Results are expressed as median, minimum and maximum values from five different GC B cell suspensions. (**B**) Flow cytometric analysis of CX_3_CR1 expression on freshly purified tonsil GC, naïve, and memory B cells. Results are expressed in box plot as median percent positive cells, minimum and maximum values, and quartiles, from ten different experiments. (**C**) Chemotaxis of GC, naïve, and memory B lymphocytes to rCX_3_CL1. Results are median numbers of migrated cells, maximum and minimum values, from five different experiments for each B cell subset. * P = 0.043 for both 300 and 600 ng/ml rCX_3_CL1. ▪ = Chemotaxis of non-GC B cells to 300 ng/ml rCXCL12 tested as control. (**D**) Freshly isolated GC B cells were pre-incubated with or without PTX and subjected to chemotaxis to 300 ng/ml CX_3_CL1 or medium (nil). Results are median numbers of migrated cells, minimum and maximum values from three different experiments.

Subsequent experiments were carried out with naïve B cells, memory B cells and B cells with a GC phenotype purified from tonsil as detailed in Material and Method. For the sake of brevity, from now onwards the latter cells will be referred to as GC B cells. Since spontaneous apoptosis of freshly isolated GC B cells was a matter of concern [10], we investigated the proportions of Annexin V^+^ early apoptotic cells at time 0 and in 1 to 4h cultures using five different GC B cell suspensions. As shown in Fig. 2A, GC viability ranged from a maximum of 96% (freshly isolated cells) to a minimum of 76% (after 4 h culture).

In ten different experiments, a half of freshly isolated GC, naïve, and memory tonsil B lymphocytes expressed CX_3_CR1, as assessed by flow cytometry (Fig. 2B). Chemotaxis to rCX_3_CL1 was next investigated in a two hour assay. In five different experiments, dose-dependent chemotaxis of GC B cells was observed (Fig. 2C). 300 and 600 ng/ml rCX_3_CL1 increased significantly chemotaxis of GC B cells compared to medium alone (P = 0.043 for both concentrations) (Fig. 2C). By contrast, naïve and memory B cells were not attracted by rCX_3_CL1, but migrated to CXCL12 tested as control (Fig. 2C).

CX_3_CR1 mediated signal transduction is inhibited by pre-treatment with pertuxis toxin (PTX) [6]. We pretreated freshly isolated GC B cells with PTX and tested their chemotaxis to rCX_3_CL1. In three different experiments, GC B cell migration was completely abolished by PTX pre-treatment (Fig. 2D).

Both IgD^+^, CD27^−^ naïve and CD27^+^, IgD^−^ memory circulating B cells expressed CX_3_CR1 (naïve B cells: median 52%, range 31–59, n = 9; memory B cells: 54%, range 24–86, n = 9) but did not migrate to rCX_3_CL1 (10–1000 ng/ml), either following stimulation with anti-human Ig plus rCD40L or after pre-culture for 6 h in medium alone [19], [20] (data not shown).

### CX_3_CR1 Mediated Signal Transduction in Tonsil B Cells

**Figure 3:**
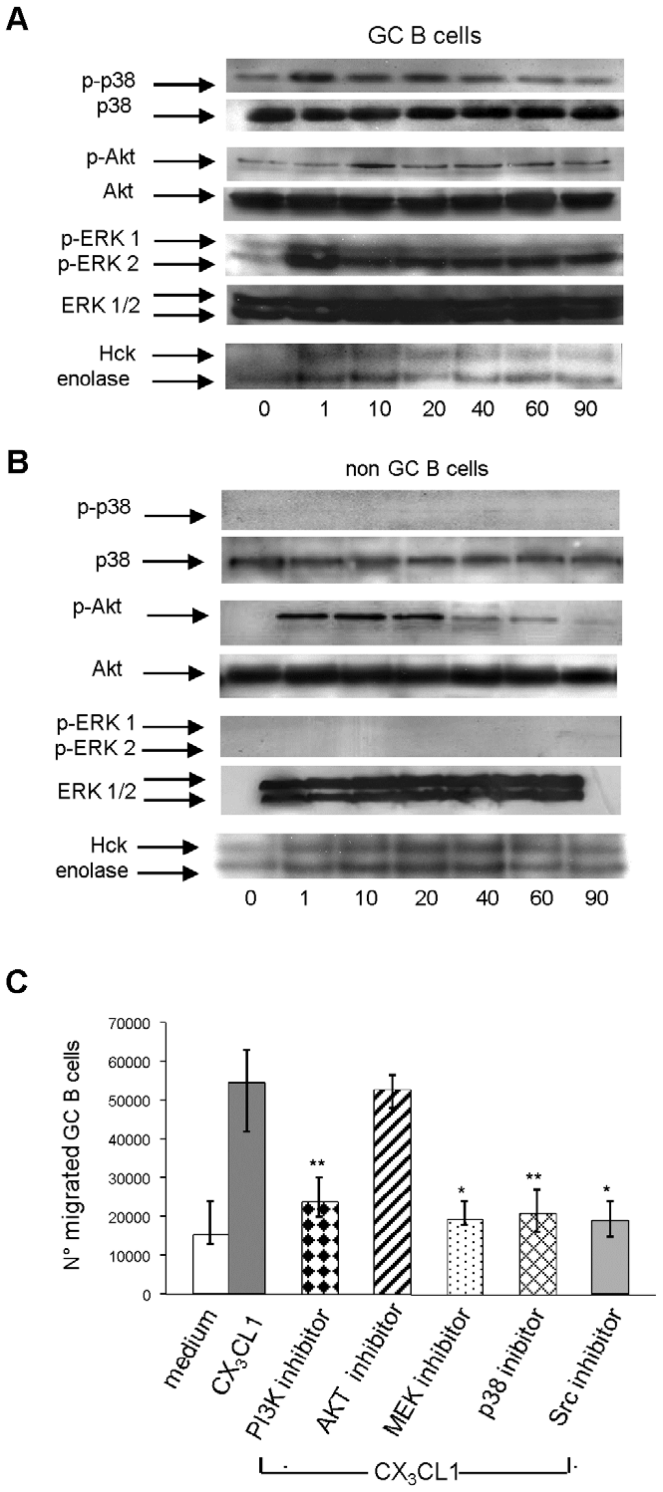
CX_3_CR1 driven signal transduction. (**A–B**) Western blot analysis of phosphorylated (p) and non-p PI3K, p38, AKT, MEK/Erk1,2 and kinase assay of Hck activity (Src family) in GC and non-GC B cells. One representative experiment out of four performed is shown. (**C**) Chemotaxis of GC B cells to 300 ng/ml rCX_3_CL1 following pre-incubation with or without the PI3K inhibitor LY294002 (20 µM), the MEK inhibitor PD98059 (1 µM), the AKT inhibitor (1 µM), the p38 inhibitor SB203580 (1 µM)or the Src inhibitor (10 µM). All cells in the bottom chamber were collected and counted. Results are median numbers of migrated cells, minimum and maximum values from five different experiments. **P = 0.009 for PI3K and p38 inhibitors; *P = 0.03 for MEK and Src inhibitors.

In four different experiments, low density CD39^−^ tonsil GC B cells [21], [22] were incubated with or without rCX_3_CL1 and subjected to Western blot analysis of MAPK phosphorylation using antibodies to phosphorylated (p)-p38, p-Akt, p-Erk1 and p-Erk2. Low levels of phosphorylated proteins were detected in freshly isolated GC B cells (Fig. 3A). Following incubation with rCX_3_CL1, p-p38 expression peaked after 1 min (mean fold increase 2.3±0,6), p-Akt after 10 min (mean fold increase 2.1±0,8), p-ERK1 after 1 min (mean fold increase 2.1±0,4), and p-ERK2 after 1 min (mean fold increase 2.2 fold±0.07). The bands corresponding to non-phosphorylated p38, Akt, Erk1 and Erk2 did not change in intensity following cell treatment with rCX_3_CL1 at any time tested (Fig. 3A). In the same cell populations, we studied Src activity by the *in vitro* kinase assay evaluating the activation of Hck, a member of Src kinases. The two bands of autophosphorylation and enolase phosphorylation peaked after 1 min rCX_3_CL1 treatment (mean fold increase 2.5±0.8 for the endogenous substrate Hck; 2.2±0.5 for the exogenous substrate enolase) (Fig. 3A).

In four different experiments, non-GC B cells incubated with soluble rCX_3_CL1 showed constitutive low level expression of p-Src, but not of p-p38, p-Akt or p-ERK 1/2 (Fig. 3B). p-Akt was induced *de novo* and peaked after 1 min exposure to rCX_3_CL1. p-p38 or p-ERK1/2 were not induced by CX_3_CL1 in non-GC B cells (Fig. 3B). In the latter cells, p-Src peaked after 1 min (mean fold increase 2.5±1.1 for the endogenous substrate Hck; 1.5±0.35 for the exogenous substrate enolase) as assessed by *in vitro* kinase assay (Fig. 3B).

Next, chemotaxis of GC B cells to CX_3_CL1 was investigated following cell preincubation with the PI3K inhibitor LY294002, Akt inhibitor, the MEK inhibitor PD98059 that targets ERK1/2, the p38 inhibitor SB203580, the Src family inhibitor PP1 or medium. GC B cell chemotaxis was significantly inhibited by the PI3K inhibitor LY294002 (20 µM) (P = 0.009), the MEK inhibitor PD98059 (1 µM) (P = 0.03), the p38 inhibitor SB203580 (1 µM) (P = 0.009) and the Src family inhibitor PP1 (10 µM)) (P = 0.03). In contrast, the Akt inhibitor had no effect (Fig. 3C).

These results demonstrate that PI3K and Src family kinases are involved in signal transduction initiated by CX_3_CL1 in human GC B cells.

### Characterization of CX_3_CR1^+^ GC B Cells

In subsequent experiments, low density CD39^−^ tonsil GC B cells were characterized immunophenotypically by double staining for CX_3_CR1 and a panel of GC B cell related markers.

**Figure 4:**
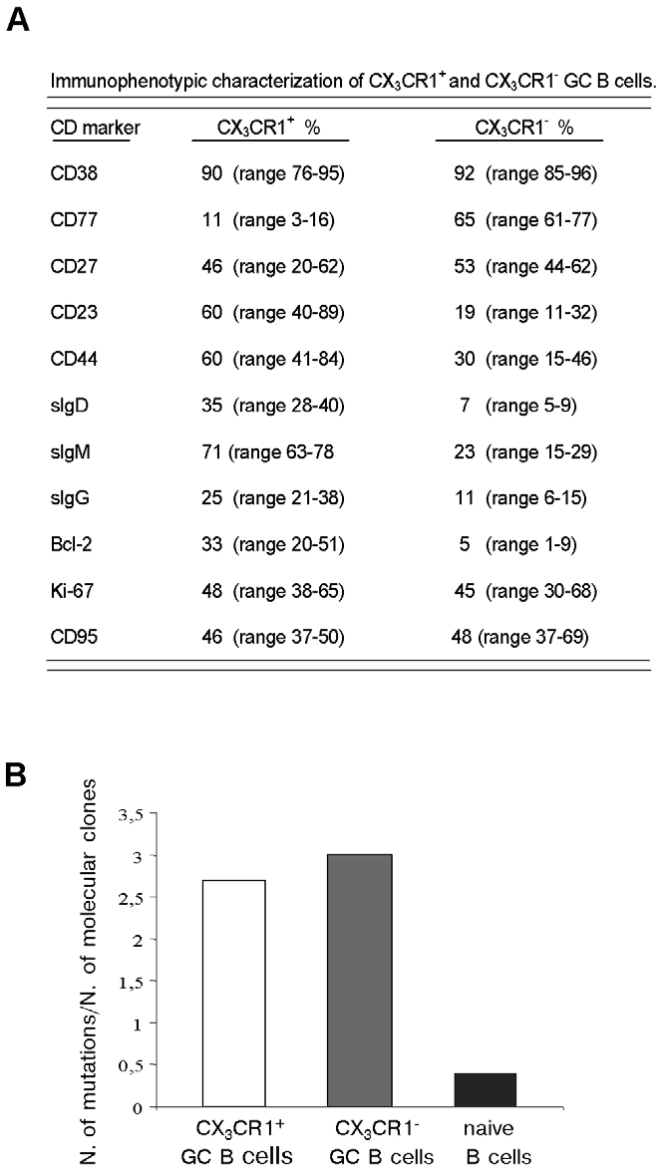
Immunophenotypic and molecular analyses of CX_3_CR1^+^ and CX_3_CR1^−^ GC B cells. (**A**) CX_3_CR1^+^ and CX_3_CR1^−^ GC B cells were analyzed after staining with mAbs. Results are percent positive cells, minimum to maximum ranges from ten independent experiments. (**B**) V_H_5(D)J_H_-μ rearrangement sequences were evaluated in CX_3_CR1^+^ and CX_3_CR1^−^ GC B cells. 104 molecular clones from five different CX_3_CR1^+^ GC B cell fractions were compared to 63 molecular clones from three different CX_3_CR1^−^ GC B lymphocyte fractions. 34 molecular clones from three different CD10^-^CD27^−^ naive B cell fractions were tested as controls. Results are ratios between number of mutations and number of molecular clones.

In ten different experiments, most CX_3_CR1^+^ GC B cells were found to express CD38, whereas the centroblast-associated marker CD77 was detected on a minority of cells (Fig. 4A). Approximately a half of CX_3_CR1^+^ GC B cells expressed CD23, CD27, CD44, Bcl-2, Ki67 and CD95 (Fig. 4A). The majority of CX_3_CR1^+^ GC B cells was surface(s)IgM^+^, while 25% expressed sIgG and 35% sIgD. Most CX_3_CR1^−^ GC B cells expressed CD38, a large fraction was CD77^+^, a half was CD27^+^, one third was CD44, whereas CD23, sIgM, sIgD, sIgG and Bcl-2 were detected in lower proportions of cells (Fig. 4A).

Morphometric analysis of cytospins from freshly isolated CX_3_CR1^+^ and CX_3_CR1^−^ GC B cells showed that the latter cells were significantly larger than the former (mean diameter ± SD = 7.4±0.97 and 6.0±0.58 micron, respectively; P = 0.003) (Supplemental Fig. 1A).

Next, CX_3_CR1^+^ and CX_3_CR1^−^ GC B cells were cultured 16 h in the presence or absence of rCD40L and IL-4 and stained with Annexin V. Both cell fractions underwent spontaneous apoptosis and were rescued by incubation with rCD40L (Supplemental Fig. 1B). In contrast, CX_3_CL1 did not affect survival of CD40L stimulated or unstimulated CX_3_CR1^+^ GC B cells in 16 h cultures.

V_H_5(D)J_H_-μ rearrangement sequences were next evaluated in CX_3_CR1^+^ and CX_3_CR1^−^ tonsil GC B cell subsets. IgVH5 gene family rearrangements were chosen because i) IgM was the major isotype expressed within CX_3_CR1^+^ GC B cells, and ii) the VH5 family is composed of a relatively small number of members (and allelic variants) thus allowing an extremely reliable mutation analysis [23], [24].

We compared 104 molecular clones expressed in CX_3_CR1^+^ GC B cells from 5 subjects to 63 molecular clones expressed in CX_3_CR1^−^ GC B lymphocytes from 3 individuals. In addition, 34 molecular clones from CD10^−^, CD27^−^ naive B cells of 3 out of the 5 subjects tested above were analyzed as controls. In both CX_3_CR1^+^ and CX_3_CR1^−^ GC B cells the majority of V_H_ sequences showed ≤1% mutations (≤3 nucleotide substitutions). In particular, 67/104 (64.4%) from CX_3_CR1^+^ GC B cells and 42/63 (66.6%) clones from CX_3_CR1^−^ GC B lymphocytes displayed <1% deviation from the V_H_ germline sequence. In V_H_5 sequences from CD10^−^, CD27^−^ naive B cells, only one clone out of the 34 analyzed showed >3 mutations (Supplemental Table 1).

The number of somatic mutations per clone was similar in CX_3_CR1^+^ and CX_3_CR1^−^ GC B cell subsets. A total of 281 mutations were observed in the 104 VH5 sequences from CX_3_CR1^+^ GC B cells (2.7 mutations per V_H_ sequence) compared to 189 mutations detected in the 63 clones from CX_3_CR1^−^ GC B cells (3.0 mutations per V_H_ sequence) (Fig. 4B). Fifteen nucleotide substitutions were found in the 34 molecular clones from CD10^−^, CD27^−^ naïve B cells (0.4 mutations per V_H_ sequence) (Fig. 4B).

Antigen selection, as determined by significant accumulation of R mutations in the complementary determining region (CDR) regions and/or significant preservation of framework (FR) amino acid sequences, was observed in similar proportions of mutated V_H_ sequences both in the CX_3_CR1^+^ and CX_3_CR1^−^ GC B cell subsets (8/35 and 4/19 clones respectively) (Supplemental Table 1).

Clonal relatedness (but not intraclonal diversification) was observed in 2 out of the 3 groups of CX_3_CR1^−^ GC B cell molecular clones, whereas it was not detected in any group of CX_3_CR1^+^ GC B cell molecular clones. Finally, no clonal relatedness between CX_3_CR1^+^ and CX_3_CR1^−^ GC B cell clones from the same subjects was observed.

### Immunophenotypic and Functional Dissection of CX_3_CR1^+^ GC B Cells

**Figure 5:**
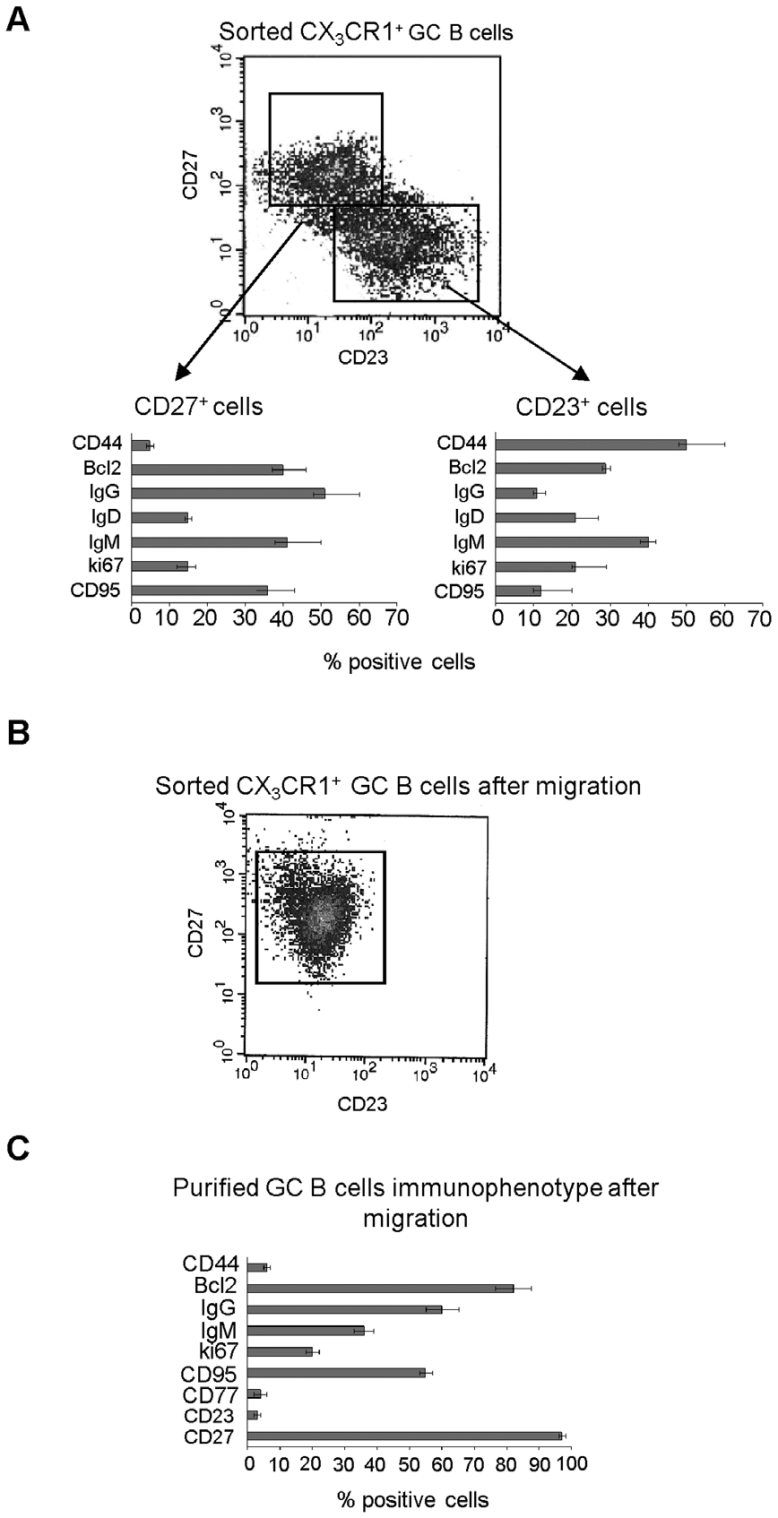
Immunophenotypic and functional dissection of CX_3_CR1^+^ GC B cells. (**A**) Double staining of CX_3_CR1^+^ GC B cells with CD27 and CD23 mAbs. Each B cell fraction was further characterized with mAbs (CD27^+^ cells, right lower panel; CD23^+^ cells, left lower panel). Results are median percent positive cells, maximum and minimum values from ten different experiments. (**B**) CX_3_CR1^+^ GC B cells were subjected to chemotaxis to 300 ng/ml rCX_3_CL1. Migrated cells were double stained for CD27 and CD23. One representative experiment out of seven is shown. (**C**) Purified GC B cells were subjected to CX_3_CL1-driven chemotaxis and migrated cells were collected and stained with a panel of mAbs. Results are median percent positive cells, maximum and minimum values from four different experiments.

The immunophenotypic profile of CX_3_CR1^+^ GC B cells indicated that these cells expressed CD23 and CD27 (Fig. 4A), that are detected in mutually exclusive B cell subsets [25], [26]. To gain further insight into this issue, CX_3_CR1^+^ GC B cells were positively selected from low density CD39^−^ GC B cells by immunomagnetic beads and double stained with CD23 and CD27 mAbs. Fig. 5A, upper panel, shows that CD23 and CD27 mAbs marked two discrete CX_3_CR1^+^ GC B cell subsets.

Next, CX_3_CR1^+^ GC B cells isolated as above were double stained with CD23 or CD27 mAbs in combination with a panel of mAbs to various GC B cell related markers.

CX_3_CR1^+^, CD23^+^ GC B cells contained CD44^+^, IgM^+^, Bcl2^+^ cells with lower proportions of cells expressing IgD, Ki-67, CD95 or IgG (Fig. 5A, right lower panel). In contrast, CX_3_CR1^+^, CD27^+^ GC B cells contained IgG^+^, IgM^+^, CD95^+^, Bcl-2^+^, CD44^−^ cells, with lower proportions of cells expressing Ki-67 or IgD (Fig. 5A, left lower panel).

CX_3_CR1^+^ GC B cells isolated as above were then subjected to chemotaxis to CX_3_CL1 and migrated cells were characterized by double staining for CD23 and CD27. Fig. 5B shows that migrated cells were comprised of CD27^+^ cells only.

In order to characterize more extensively the immunophenotype of GC B cells attracted by CX_3_CL1, low density CD39^−^ GC B cells were subjected to CX_3_CL1-driven chemotaxis and migrated cells were subsequently stained with a panel of mAbs to GC B cell related markers. As shown in Fig. 5C, most of the GC B cells attracted by CX_3_CL1 had a CD27^+^, Bcl-2^+^, IgG^+^, CD77^−^, CD44^−^, CD23^−^ immunophenotype, approximately a half expressed IgM and CD95, a minority of cells was ki67^+^. These experiments demonstrate that the GC B cells attracted by CX_3_CL1 displayed the immunophenotypic features of centrocytes.

### Expression of CX_3_CL1 in Tonsil Cell Populations

**Figure 6:**
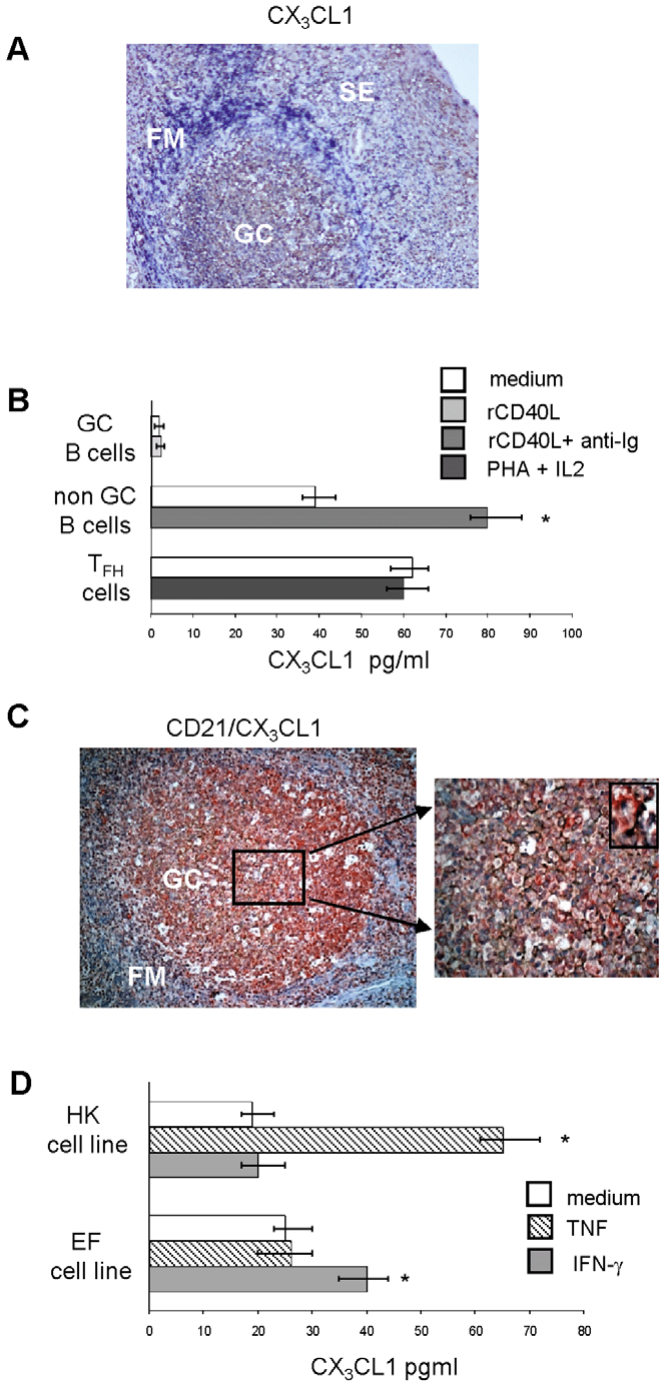
CX_3_CL1 expression in tonsil germinal center populations. (**A**) Formalin-fixed tonsil tissue section was stained with anti-CX_3_CL1 mAb (brown). GC, germinal center; FM, follicular mantle, SE, subepithelial area. One representative experiment out of three is shown. (**B**) GC B cells, non-GC B cells and T_FH_ cells were tested for CX_3_CL1 production in 24h culture supernatants by ELISA. GC B cells were cultured with or without rCD40L, non-GC B cells with or without anti-Ig antibody plus rCD40L, and T_FH_ cells with or without PHA plus IL-2. Results are median pg/ml CX_3_CL1, minimum and maximum values from four different experiments.* P = 0.028. (**C**) Double staining of formalin-fixed tonsil tissue section for CX_3_CL1 (red) and CD21 (brown). Individual double positive cells are shown in the small right panel. (**D**) CX_3_CL1 production by human FDC cell lines HK and EF cultured 24 h without or with TNF (10 µg/ml) or IFN-γ (10 µg/ml). Results are median pg/ml CX_3_CL1, minimum and maximum values from four different experiments. *P = 0.026.

CX_3_CL1 expression was investigated in tonsil tissue sections by immunohistochemistry. As shown in Fig. 6A (one representative experiment out of three), CX_3_CL1 was expressed in the GC, the follicular mantle (FM) and the subepithelial area (SE).

Next, GC B cells, non-GC B cells and CD4^+^, CXCR5^+^ T_FH_ cells were tested for production of soluble CX_3_CL1 in 24 h culture supernatants. GC B cells were cultured with or without rCD40L, non-GC B cells with or without anti-Ig antibodies plus rCD40L, and T_FH_ cells with or without PHA plus IL-2 [27]. As shown in Fig. 6B, GC B cells did not release CX_3_CL1 irrespective of the culture conditions, non-GC B cells produced constitutively soluble CX_3_CL1 and such production increased significantly (P = 0.028) following stimulation, while the amounts of CX_3_CL1 released by unstimulated and stimulated follicular helper T cells were similar.

Expression of CX_3_CL1 by FDC was subsequently investigated by immunohistochemical staining of formalin-fixed tonsil sections with CD21 and anti-CX_3_CL1 mAbs. FDC were found to express CX_3_CL1 (Fig. 6C).

We next investigated CX_3_CL1 release in 24 h culture supernatants from two human FDC cell lines, i.e. HK cells [28] and EF, generated in our lab [29]. Both cell lines were incubated without or with TNF or IFN-γ, two physiological inducers of CX_3_CL1 production [30]. Fig. 6D shows that, in four different experiments, constitutive release of CX_3_CL1 by HK and EF cells was increased significantly upon stimulation with TNF for the HK cell line (P = 0.028) and with IFN-γ for the EF cell line (P = 0.026).

### Impaired Production of Specific IgG in CX_3_CR1^−^/^−^ and CX_3_CL1^−^/^−^ Immunized with OVA

**Figure 7:**
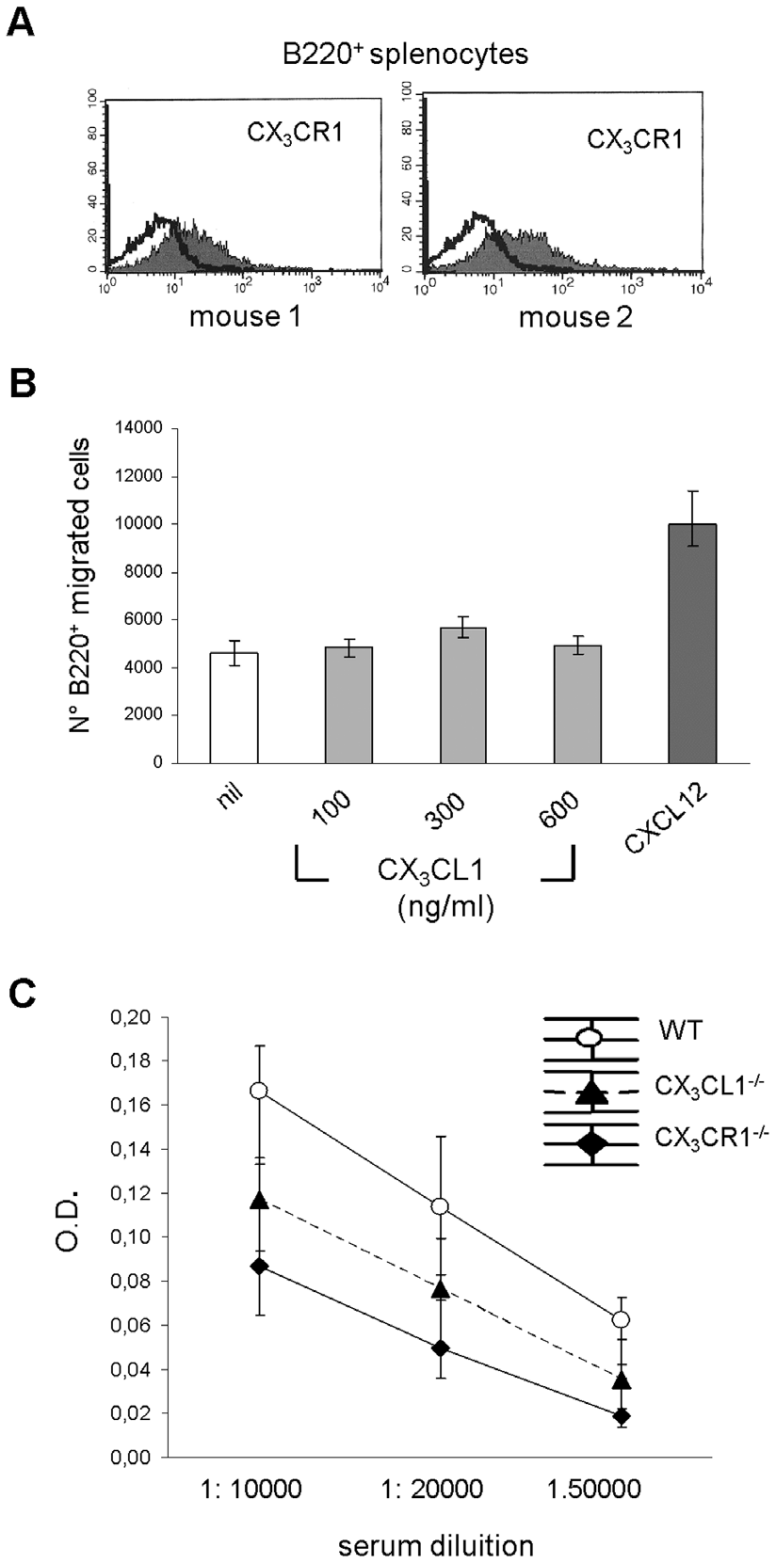
Impaired OVA specific IgG production in CX_3_CR1^−^/^−^ or CX_3_CL1^−^/^−^mice. (**A**) Splenocytes from WT mice double stained with anti-CX_3_CR1 and B220 mAbs were analyzed by flow cytometry. Histograms from two different WT mice are shown. (**B**) Splenocytes from WT mice were tested for chemotaxis to murine rCX_3_CL1 or rCXCL12 (control). Migrated B cells were enumerated by flow cytometry using anti-B220 mAb. Results are median number of migrated cells, minimum and maximum values from four different experiments. (**C**) CX_3_CR1^−^/^−^, CX_3_CL1^−^/^−^ or WT mice were immunized with OVA and tested at different dilutions for specific IgG production by ELISA. Results were expressed as median optical densities (OD), maximum and minimum values, from ten different experiments. Serum from two WT mice was titrated for OVA IgG antibodies (1: 200, 1:500, 1:1000, 1:10000, 1:50000, 1:100000 dilutions) and used in each tests as standard curve, whereas serum from mice before immunization was used as negative control.

First, splenocytes from WT mice were double stained with the pan-B anti-B220 mAb and anti-CX_3_CR1 antiserum. As shown in the representative experiments in Fig. 7A, a large proportion of splenic B cells expressed CX_3_CR1.

Splenocytes from WT mice were next tested for chemotaxis to murine CX_3_CL1 and CXCL12 as control. Migrated B cells were enumerated by flow cytometry using anti-B220 mAb. As shown in Fig. 7B, no migration was detected in response to CX_3_CL1, whereas CXCL12 induced B cell chemotaxis.

Finally, CX_3_CR1^−^/^−^ or CX_3_CL1^−^/^−^ genes [31], [32] or WT mice were immunized with the T cell dependent antigen OVA and tested for production of specific IgG. Both CX_3_CR1^−^/^−^ and CX_3_CL1^−^/^−^ mice produced significantly lower concentrations of specific serum IgG (P = 0.0002) than WT mice upon OVA immunization (Fig. 7C). Spleens from CX_3_CR1^−^/^−^ and CX_3_CL1^−^/^−^ and WT immunized mice did not differ in the microarchitecture of lymphoid follicles or the proportions or distribution of T (CD3^+^) and B (B220^+^) cells. Likewise, the proportions of T_FH_ did not differ in CX_3_CR1^−^/^−^ or CX_3_CL1^−^/^−^ *vs* WT mice (mean percent positive cells 4% for CX_3_CR1^−^/^−^ mice, n = 7; 4.2% for CX_3_CL1^−^/^−^ mice, n = 7; 5% for WT mice, n = 7) as assessed by flow cytometry [33].

These results indicate that impaired IgG production in CX_3_CR1^−^/^−^ or CX_3_CL1^−^/^−^ mice was related to deficient interaction between surface CX_3_CR1 and CX_3_CL1, but do not allow to establish whether this effect is attributable to direct ( B cell related) or indirect (accessory cell related) mechanisms.

## Discussion

Human GC B cells migrate *in vitro* to anti-Ig antibodies [17] or CXCL12 [18] only following CD40 mAb and IL-4 mediated rescue from apoptosis. Murine GC B cells overexpressing Bcl-2 under control of the Ig μ enhancer and therefore resistant to spontaneous apoptosis show different homing properties regulated by chemokine gradients *in vivo*. In particular, centroblasts express high levels of CXCR4 and home to the dark zone where the CXCR4 ligand CXCL12 is abundantly expressed. Centrocytes expressing high levels of CXCR5 accumulate in the light zone of the GC where the CXCR5 ligand CXCL13 is expressed at elevated concentrations [11]. Thus, current evidence indicates that only GC B cells rescued from apoptosis are enabled to migrate along chemokine gradients.

Here we show unambiguously that human B cells expressed CX_3_CR1 mRNA and protein as assessed by quantitative PCR, flow cytometry and competition binding. The discrepancy between these and previous results is likely related to differences in the antibodies and the techniques for mRNA analysis used [6], [8], [9].

CX_3_CR1 triggered GC B cell chemotaxis to CX_3_CL1, that was abolished by PTX pre-treatment, as expected from the well known PTX sensitivity of CX_3_CR1[6]. Signal transduction induced by CX_3_CL1 in GC B cells was similar to that previously reported for other cell types [34], [35]. In particular, p-PI3K, p-Erk1/2, p-p38 and p-Akt, as well as p-Src, were found to be up-regulated in CX_3_CL1 treated *vs* untreated GC B cells. Inhibition of PI3K, Erk1/2, p38 or Src, but not of Akt, suppressed CX_3_CL1 induced GC B cell migration. Thus, the PI3K and Src pathways integrated signals originating from either scaffold, as reported for other GPCR [36], [37].

CX_3_CR1^+^ naïve and memory B cells were not attracted by CX_3_CL1 and showed a limited signalling pattern, i.e. p-Akt and p-Src were up-regulated, whereas p-PI3K, p-p38, and p-ERK1/2 were not. These results indicate that i) signalling and chemotaxis are dissociated in non-GC B cells, ii) lack of p-PI3K activation is consistent with the crucial role of this pathway in GC B cell migration, and iii) CX_3_CL1 driven signalling in non-GC B cells suggests functions of the chemokine alternative to chemotaxis that warrant investigation.

CX_3_CR1^+^ GC B cells were almost devoid of CD77^+^ centroblasts and enriched for cells expressing the naïve B cell-related markers CD23, Bcl-2, sIgM and sIgD compared to CX_3_CR1^-^ counterparts. The proportions of Ki-67^+^ proliferating cells was similar in CX_3_CR1^+^ and CX_3_CR1^-^ GC B cells, challenging the tenet that proliferation in the GC is restricted to centroblasts [13].

Analysis of V_H_5(D)J_H_-^μ^ rearrangement sequences demonstrated that the rate of somatic mutations was similar in CX_3_CR1^+^ and CX_3_CR1^−^ GC B cells, a finding consistent with the similar expression of CD27 [25]. Likewise, the proportions of antigen selected CX_3_CR1^+^ and CX_3_CR1^−^ GC B cells was superimposable. Lack of clonal relatedness between these cell populations may depend on the relatively limited size of the samples investigated.

CX_3_CR1^+^ GC B cells contained two major subsets expressing CD23 or CD27. CD23^+^ cells contained CD44^+^, IgM^+^ and Bcl2^+^ cells, that may represent antigen-activated naïve B cells that have recently accessed the GC [38]. CD27^+^ cells were enriched for cells expressing IgG, IgM, Bcl2 and CD95 but not CD44. Only the CX_3_CR1^+^, CD27^+^ GC B cell subset was attracted by CX_3_CL1. Further characterization of migrated GC B cells showed that they displayed a predominant CX_3_CR1^+^, CD27^+^, Bcl-2^+^, IgG^+^, CD77^−^, CD44^−^, CD23^−^ immunophenotype, consistent with that of centrocytes. Therefore, these experiments suggest that CX_3_CL1 participates in the control of centrocyte trafficking within the light zone of the GC.

In the GC, T_FH_ cells promote centrocyte differentiation to Ig-secreting cells [33], and contact between centrocytes and FDC results into positive selection of centrocytes expressing high affinity B cell receptors for antigen displayed on FDC [10]. Recent studies have suggested that FDC and T_FH_ cells may collaborate in B cell selection. Thus, B cells that have captured, processed and presented high amounts of antigen to T cells would receive help from the latter cells at the expenses of B cells that have captured lower concentrations of antigen [13].

In this study, CX_3_CL1 was abundantly expressed in the GC, as assessed by immunohistochemistry [2]. Such expression was detected not only in the GC, but also in the FM and SE area. These findings are consistent with our unpublished results (Corcione A et al.) showing that a half of GC, naïve and memory B cells express constitutively CX_3_CL1 on the cell surface.

CX_3_CL1 was released constitutively by T_FH_ cells and FDC, whose production was increased by stimulation with TNF or IFN-γ [30], two cytokines produced in tonsils *in vivo* [39]. In this respect, our preliminary experiments show that T_FH_ cells and FDC express constitutively ADAM10, a metalloprotease involved in constitutive shedding of CX_3_CL1 from the cell surface [40]. Since T_FH_ cells and FDC reside in the light zone of GC, they may control through CX_3_CL1 release local *in vivo* migration of human CX_3_CR1^+^ centrocytes. Thus, CX_3_CL1 would cluster together T_FH_ cells, FDC and centrocytes and promote differentiation of the latter cells to Ig-secreting cells.

Mouse B cells, although expressing CX_3_CR1, did not migrate to CX_3_CL1, indicating that surface rather than soluble CX_3_CL1 may be involved in cellular interactions leading to antigen specific IgG production.

*In vivo* experiments demonstrated that OVA-specific IgG production was significantly impaired in both CX_3_CR1^−^/^−^ or CX_3_CL1^−^/^−^ mice, in the absence of changes in microarchitecture, organization of lymphoid follicles and lymphocyte subset distribution. Further studies are required to understand what mechanisms underlie defective antibody production and where they operate, i.e. in secondary lymphoid organs or at periphery.

In conclusion we have identified a novel chemokine candidate to regulate human centrocyte trafficking in secondary lymphoid organs. Differences between human and mouse models highlight the difficulties at comparing the results obtained in these systems.

## Materials and Methods

### Ethic Statement

For research involving human participants, in our Institute this type of study did not require an ethic statement prior to the use because all tonsil samples are generally discarded after surgery and peripheral blood samples are from healthy donors of Blood Transfusion Center of the Institute. However, an informed consent, performed at the Medical Direction (Legal Medicine) of the G.Gaslini Institute, was asked to children parents or their legal guardians for tonsil samples. As well as, an informed consent was asked to donator for blood samples. All procedures involving animals were performed in the respect of the National and International current regulations (D.l.vo 27/01/1992, n.116, European Economic Community Council Directive 86/609, OJL 358, Dec. 1, 1987).

### Antibodies

The following mAbs were used: CD19-PE/cyanin (Cy)5, CD38-PE/Cy5 (HIT2 clone), CD23-PE, CD4-PE/Cy5, and CD4-FITC from Caltag Laboratories (Burlingame, CA, USA); CD38-PE/-FITC and goat anti-rabbit Ig-PE/FITC from Serotec Inc. (Raleigh, NC, USA); CD27-PE/-FITC, anti-CXCR5-PE, and anti-IgG-FITC mAbs from BD Pharmingen (San Diego, CA, USA). CD10-FITC (MEM78 clone), anti-human IgD-FITC, CD95-FITC, CD44-FITC, anti-Bcl-2-FITC, and anti-Ki-67-FITC mAbs from DAKO (Glostrup, Denmark); unconjugated CD77 and CD39 mAbs from Immunotech, Marseille, France; unconjugated rabbit anti-human CX_3_CR1 (Serotec) that detects an epitope starting from 175 to 189 amino acids; unconjugated CD3, CD56, CD68, CD10, CD27, and anti-human IgM mAbs from DAKO. Rat anti-mouse CD4-PE/Cy5.5, rat anti-mouse CXCR5-PE, rat anti-mouse CD19-FITC, rat anti-mouse CD3-FITC, and hamster anti-mouse CD128-FITC were from BioLegend (San Diego, CA, USA).

Cells were stained with fluorochrome conjugated or unconjugated antibodies followed by secondary reagents. Isotype and fluorochrome matched antibodies were tested as controls. For intracellular staining, cells were fixed, permeabilized, incubated with fluorochrome-conjugated mAbs and analyzed. Cells were run on a FACSCalibur (BD). 10^4^ events were acquired and analyzed using the CellQuest software (BD).

### Cell Isolation and Culture

Mononuclear cells (MNC) were isolated from tonsils and peripheral blood by Ficoll-Hypaque (Sigma Chemical Company, St. Louis, MO) and depleted of T lymphocytes by rosetting with sheep erythrocytes and of CD56^+^ and CD68^+^ cells by immunomagnetic beads. (Milteny Biotec Inc., Auburn, CA). These fractions contained >95% CD19^+^ B cells.

Tonsil B lymphocytes were incubated with CD10 mAb and separated by immunomagnetic beads into CD10^+^ GC [41] and CD10^−^ non-GC B cells at 4°C to prevent spontaneous apoptosis. The latter cells were further separated into CD27^+^ memory and CD27^−^ naïve B cells by immunomagnetic beads [25], [42]. Alternatively, GC B cells were isolated from the low density 30/40 fraction of a Percoll gradient (Pharmacia, Uppsala, Sweden) [21], [22] followed by depletion of CD39^+^ B cells. In some experiments, CD39^−^ low density B cells, that contained >95% CD10^+^ GC B lymphocytes, were separated into CX_3_CR1^+^ and CX_3_CR1^−^ cells. Annexin V staining (Bender Systems, Burlingame, CA, USA) was used to detect apoptosis.

CD10^+^ GC B cells were cultured 24 h with or without rCD40L (100 ng/ml) (Immunotools). CD10^−^ non-GC B cells were cultured 24 h with or without rCD40L and goat anti-human Ig antibodies (2 µg/ml) (Jackson Immunoresearch, CA, USA). Supernatants were stored at −80°C.

Tonsil T cells were incubated 30 min at 4°C with CD4 FITC mAb and anti-CXCR5 PE mAb. CD4^+^CXCR5^+^ T follicular helper_ (FH)_ cells [33] were sorted (98% purity) by FACSAria (BD). T _FH_ cells were cultured 24 h with or without PHA (1 µg/ml) (MP Biomedicals, Ca, USA) and IL-2 (50 U/ml) (Proleukin, Chiron Italia, Milan Italy) [27]. Supernatants were stored at −80°C.

### Chemotaxis

Chemotaxis was investigated using 5 µm pore-size transwell plates (Costar, Cambridge, MA). Five ×10^5^ cells were dispensed in the upper chamber, and increasing concentrations of rCX_3_CL1/Fractalkine (R&D System) or medium were added to the lower chamber. rCXCL12 (R&D System) was tested as control in some experiments at the concentration of 300 ng/ml [18].

GC B cells were pre-treated 1 h at 37°C with 100 ng/ml PTX (Sigma) and subjected to chemotaxis to rCX_3_CL1. GC B cells were pre-incubated 1 h with PI3K inhibitor LY294002 (20 µM; Sigma), Akt inhibitor (1 µM; Calbiochem, Gibbstown, NJ. USA), MEK inhibitor PD98059 (1 µM; Biomol, Plymouth, Meeting, PA, USA), p38 inhibitor SB203580 (1 µM; Biomol), Src family inhibitor PP1 (10 µM; Calbiochem) or medium before undergoing chemotaxis. Plates were incubated 2 h at 37°C. Migrated cells were collected and counted.

Mouse splenocytes were tested for chemotaxis to murine rCX_3_CL1 (100–600 ng/ml) or mCXCL12 (300 ng/ml) (both from R&D System). Migrated B cells were enumerated by CD19 staining.

### Follicular Dendritic Cell Line Isolation and Culture

Two human FDC lines, HK cells [28] and EF cell generated in our lab [29], were cultured 24 h with or without TNF (10 µg/ml, R&D System) or IFN-γ (10 µg/ml, R&D System) and supernatants stored at −80°C.

### Signal Transduction Assays

Western blot analysis of MAPK phosphorylation and kinase assay of Hck activity (Src family) were investigated in GC and non-GC B cells and performed as reported [43]. Cells were incubated with or without 300 ng/ml rCX_3_CL1 for 1, 10, 20, 40, 60, and 90 min. The pellets were lysated with NP40 buffer. In the Western blot assay, lysates resolved by gel electrophoresis, transferred to nitrocellulose membranes, and incubated with specific anti p-p38, anti p-Akt, and anti p-ERK-1/2 mAbs (all from Santa Cruz Biotechnology). The membranes were stripped with Restore^TM^ Western Blot Stripping Buffer (Pierce, Rockford, IL, USA) and re-probed with mAbs to detect total p38, Akt, and ERK-1/2 (Santa Cruz Biotechnology). After incubation with horse radish peroxidase (HRP) antibodies, blots were developed with Enhanced Chemiluminescence Detection. In kinase assay, lysates (300 µg) were pre-cleared with rabbit serum and Protein A-agarose immunoprecipitated overnight at 4°C with 1 µg Hck mAb (Santa Cruz Biotechnology, Santa Cruz, CA, USA). Each immunoprecipitate sample was washed twice with lysis buffer and twice with kinase buffer (30 mM HEPES, pH 7.5, 10 mM MgCl_2_, 1 mM CaCl_2_, 5 mMCl_2_). After washing, cells were incubated for 15 minutes at 30°C in 30 µl kinase buffer 10 µM ATP (Sigma), and 5 µCi γ^32^P ATP (ICN Biomed) in presence or in absence of 10 µg of acid-denaturated enolase (muscle rabbit enolase, Sigma, St. Louis, MO, USA). The reaction was stopped by the addition of 4X Laemmli sample buffer and boiled for 5 min. Samples were separated by electrophoresis on 12% SDS-polyacrylamide gel. Kinase activity was detected by autoradiography.

### Densitometric Analyses

Immage of immunoblots and kinase assay were scanned and quantification was carried out by IAS 2000 Image Analysis program from Deltasistemi (Latina, Italy). For MAPKs phosphorylation, values were normalized to total amounts of p38, Akt, and ERK-1/2. Ratios between values of the most intense band following rCX_3_CL1 treatment and those at time 0 are expressed as mean fold increase±SEM from four independent experiments.

### Competition Binding Assay

The competition binding assay was performed according to Wang et al. [44]. Briefly, cells were suspended in binding buffer (PBS pH 7.4 containing 2% BSA) supplemented with 1 nM ^125^I-CX_3_CL1 (Perkin Elmer, specific activity 2200/Ci mmol) in the absence or presence of 1, 10 and 100 nM cold CX_3_CL1 for 2 h at 4°C. Non specific binding was determined in parallel through cell incubation with ^125^I-CX_3_CL1 in the presence of 100-fold excess unlabeled CX_3_CL1 and subtracted from total binding to yield specific binding. After incubation, medium was removed, cells were washed with binding buffer and radioactivity counted. Percentage of binding inhibition by unlabeled CX_3_CL1, calculated as ratio between cell-bound cpm in the presence of unlabeled ligand and cell-bound cpm in the absence of unlabeled ligand multiplied by 100, was used as a measure for competition between ^125^I-labeled and unlabelled CX_3_CL1. Some experiments were performed using 100 nM cold CXCL8 (BioSource International, Flynn, Camarillo, Ca, USA) as negative control.

### Amplification and Sequencing of the Ig V_H_5(D)J Rearrangements Derived from Tonsil B Cell Subsets

DNA-free RNA was extracted using the RNeasy mini kit (Qiagen S.p.A., Milan, Italy). One µg RNA was reverse-transcribed into cDNA using M-MLV reverse transcriptase (RT, Invitrogen S.R.L., Milano, Italy) primed by an oligo dT (16mer) primer. Reactions were carried out in 25 µl volume using 20 pmoles of primer, 200 U of RT at 42°C for 1 h. IgV_H_5 rearrangements were determined by amplifying 2 µl of cDNA with primers and PCR conditions previously described [23], [24]. Sequences were compared with Ig germline gene database at ImMunoGeneTics ® http://imgt.cines.fr [45]. MacVector 6 software (Accelrys, S.Diego, CA, USA) was used for further sequence analyses.

Distribution of mutations among CDR and FR gene segments was evaluated by the Chang–Casali binomial distribution model [46]. A binomial probability model was used to evaluate whether the excess of replacement (R) mutations in CDR or their scarcity in FR was due to chance.

### mRNA Analysis by Quantitative RT-PCR

RNA extraction and reverse transcription were performed as above. CX_3_CR1-R FW 679 TTTTCTTGGCTTCCTACTCC, CX_3_CR1-R RV 809 CACGATGACCACCAGAAGG primers and a TaqMan MGB probe (5′-YAK- TGTGGTTCTTGCAGGAAAACAGC-BBQ-3′) were designed. Quantification of CX_3_CR1 mRNA expression was performed on a Rotor-Gene 3000 instrument (Corbett Life Science, Sydney, Australia). Two independent experiments, with samples run in quadruplicate, were performed. As endogenous control, polymerase II polypeptide A (*POLR2A*) was co-amplified in the reaction using a FAM labeled probe. The relative expression of CX_3_CR1-R among the samples was calculated using the ΔΔCt method as implemented in the Rotorgene 3000 software (Corbett Life Science). CX_3_CR1 expression was normalized among samples using the *POLR2A* expression level and was represented as fold of CX_3_CR1 expression relative to the THP-1 cell line, arbitrarily set at 1.

### Immunohistochemistry

Immunohistochemical staining of tonsil tissue sections was performed using the Envision System HRP and alkaline phosphatase (AP) mouse (DAKO), as reported [47]. In double staining experiments, sections were incubated in DAKOCytomation Antigen Retrieval Solution for antigen retrieval, and subsequently with CD21 mAb (DAKO). The slides were then incubated with Dako Envision System HRP Mouse and peroxidase activity was detected with DAKO Liquid DAB Substrate Chromogen System. Then, the slides were washed, incubated overnight at 4C° with anti-CX_3_CL1 mAb (PeproTech, Rocky Hill, NJ, USA) and treated with DAKO Envision System AP mouse (DAKO). AP activity was detected by incubating the sections for 20 min at room temperature with DAKO Liquid Fucsin Substrate Chromogen System (DAKO) and counterstained with Mayer's hematoxylin (Sigma; St. Louis, MO, USA).

Formalin fixed, paraffin embedded mouse tissue sections were incubated for 60 min at room temperature with a rat anti-mouse B220 (clone RA3-6B2, Serotec) or a rat anti-mouse CD3 (Abcam, Cambridge; UK). Sections were subsequently reacted for 30 min at room temperature with HRP conjugated rabbit polyclonal antibodies to rat IgG (Abcam). Controls were sections incubated in the absence of primary antibody or with irrelevant primary antibodies of the same isotype as test mAb.

### In Vivo Studies

Twenty wild type (WT) mice (B6), 15 CX_3_CR1^−^/^−^ gene (6V28) and 20 CX_3_CL1^−^/^−^ (6FK) were immunized intra-peritoneally on days 1 and 8 with 50 µg (100 µl) OVA (Sigma) or PBS in Alum solution (Pierce Chemical Company, Rockford, IL, USA) [48]. After two weeks mice were sacrificed. Sera were collected before immunization and at sacrifice. To quantitate OVA specific IgG antibodies, 96 well plates were coated overnight with 10 µg/ml OVA, washed with PBS plus 0.05 Tween (Sigma) and blocked with PBS plus 10% FBS. 100 µl serum samples were incubated for 2 h at room temperature at the 1∶10000, 1∶20000, and 1∶50000 dilutions. Plates were washed three times, incubated with 100 µl of anti mouse IgG-HRP antibody (1∶5000 dilution) for 45 min at room temperature. Reaction was developed with the HRP substrate tetramethyl benzidine (TMB) (Becton-Dickinson). Results were expressed as optical densities at 405 nm. Sera from two OVA immunized WT mice were titrated for OVA IgG antibodies (1: 200, 1∶500, 1∶1000, 1∶10000, 1∶50000, 1∶100000 dilutions) and used in each tests as standard curves, whereas sera from mice before immunization were used as negative controls.

### Statistical Analysis

Data were reported in terms of medians, minimun and maximum values or quartiles. The Mann-Whitney U test was used to compare quantitative variables between two groups of observation. The one-way non parametric ANOVA (Kruskal Wallis test) was used for *in vivo* experiments to compare parameters in more than two groups of data. The Mann-Whitney U test with the Bonferroni's correction was used as a *posterior* test. A *P* value of less than 0.05 was considered statistically significant. Statistical analyses were performed using Graph Pad Prism 3 software and the statistical package “Statistica 6”.

## Supporting Information

Figure S1 Morphological and functional characterization of CX_3_CR1^+^ and CX_3_CR1^−^ GC B cells
(3.67 MB DOC)

Table S1 IgVH sequencing in CX_3_CR1^+^ and CX_3_CR1^−^ tonsil GC B cells
(0.30 MB DOC)
