# Correction: CX_3_CR1 Is Expressed by Human B Lymphocytes and Mediates CX_3_CL1 Driven Chemotaxis of Tonsil Centrocytes

**DOI:** 10.1371/annotation/50da3cf8-38cb-4985-8875-e41aa03191ba

**Published:** 2010-01-26

**Authors:** Anna Corcione, Elisa Ferretti, Maria Bertolotto, Franco Fais, Lizzia Raffaghello, Andrea Gregorio, Claudya Tenca, Luciano Ottonello, Claudio Gambini, Glaucia Furtado, Sergio Lira, Vito Pistoia

The word "Mediates" was misspelled in the article title. The correct title is: CX₃CR1 Is Expressed by Human B Lymphocytes and Mediates CX₃CL1 Driven Chemotaxis of Tonsil Centrocytes. The correct citation is: Corcione A, Ferretti E, Bertolotto M, Fais F, Raffaghello L, et al. (2009) CX₃CR1 Is Expressed by Human B Lymphocytes and Mediates CX₃CL1 Driven Chemotaxis of Tonsil Centrocytes. PLoS ONE 4(12): e8485. doi:10.1371/journal.pone.0008485

