# Supplementary material for: CX3CR1 Is Expressed by Human B Lymphocytes and Meditates CX3CL1 Driven Chemotaxis of Tonsil Centrocytes
Source: PLoS One. 2009 Dec 29;4(12):e8485. doi: 10.1371/journal.pone.0008485 (PMC2793522; doi:10.1371/journal.pone.0008485)
Supplement: Figure S1 — Morphological and functional characterization of CX3CR1+ and CX3CR1− GC B cells (3.67 MB DOC) [file pone.0008485.s001.doc]

**Supplemental Fig. 1. Morphological and functional characterization of CX_3_CR1^+^ and CX_3_CR1^-^ GC B cells**. (**A)** Morphometric analysis of freshly isolated CX_3_CR1^+^  and CX_3_CR1^-^ GC B cell cytospins. One representative experiment out of three is shown. (**B)** Purified CX_3_CR1^+^ and CX_3_CR1^-^ GC B cells were cultured 16 h with or without rCD40L. Apoptosis was assessed by Annexin V staining. Results are median percent Annexin V^+^ cells, maximum and minimum values.
