# Supplementary material for: CX3CR1 Is Expressed by Human B Lymphocytes and Meditates CX3CL1 Driven Chemotaxis of Tonsil Centrocytes
Source: PLoS One. 2009 Dec 29;4(12):e8485. doi: 10.1371/journal.pone.0008485 (PMC2793522; doi:10.1371/journal.pone.0008485)
Supplement: Table S1 — IgVH sequencing in CX3CR1+ and CX3CR1− tonsil GC B cells (0.30 MB DOC) [file pone.0008485.s002.doc]

**Supplemental Table 1. IgVH sequencing in CX_3_CR1^+^ and CX_3_CR1^-^ tonsil GC B cells**

|  | TONS 1  CX3CR1+ | IgVH gene | % of IgVH identity | N. of IgVH mutations | CDR3 aminoacid composition |
| --- | --- | --- | --- | --- | --- |
|  |  |  |  |  |  |
| 1 | CL. 1 | IGHV5-51 | 98.95 | 3 | C ARAPPYGSGSYLDY W |
| 2 | CL. 3 | IGHV5-51 | 98.95 | 3 | C ARHAHQVVTYYYGMDV W |
| 3 | CL. 4 | IGHV5-51 | 99.30 | 2 | C ARLVVPAAFDC W |
| 4 | CL. 5 | IGHV5-51 | 100 | 0 | C ARHYYDSSGHHDAFDI W |
| 5 | CL. 6 | IGHV5-51 | 98.95 | 3 | C ARRGAAYSGSYGGGLVDY W |
| 6 | CL. 7 | IGHV5-a | 98.26 | 5 | C ARAEYYGSGSYYARFDY W |
| 7 | CL. 8 | IGHV5-51 | 98.95 | 3 | C ARHQLPYDFWRGYYPY W |
| 8 | CL. 9 | IGHV5-51 | 98.26 | 5 | C LIGSYDLYAMDV W |
| 9 | CL. 11 | IGHV5-51 | 99.65 | 1 | C ARHVGHGGYSYFDY W |
| 10 | CL. 13 | IGHV5-51 | 98.26 | 5 | C ARGARLRFLPDGYDI W |
| 11 | CL. 14 | IGHV5-51 | 100 | 0 | C ARITMVRGVITYFDY W |
| 12 | CL. 15 | IGHV5-51 | 99.65 | 1 | C ARPDYGSNAGMDV W |
| 13 | CL. 16 | IGHV5-51 | 98.26 | 5 | C AAYSSSRAFGMDV W |
| 14 | CL. 17 | IGHV5-a | 96.87 | 9 | C ARHGSYCGGSVCYPYYFDD W |
| 15 | CL. 18 | IGHV5-51 | 99.65 | 1 | C ARHAIPVANRNYFDP W |
| 16 | CL. 19 | IGHV5-a | 100 | 0 | C ARPRVGIGEDAFDI W |
| 17 | CL. 20 | IGHV5-a | 97.56 | 7 | C ARVTAAHDAFDI W |
| 18 | CL. 21 | IGHV5-51 | 98.26 | 5 | C AAYSSSRAFGMDV W |
| 19 | CL. 22 | IGHV5-51 | 100 | 0 | C ARLGITGTHAFDI W |
| 20 | CL. 23 | IGHV5-51 | 98.61 | 4 | C ARRHDYGDYSYY W |
| 21 | CL. 24 | IGHV5-a | 100 | 0 | C ARHPTVRNFDY W |

|  | TONS 2  CX3CR1+ | IgVH gene | % of IgVH identity | N. of IgVH mutations | CDR3 aminoacid composition |
| --- | --- | --- | --- | --- | --- |
|  |  |  |  |  |  |
| 1 | CL. 1 | IGHV5-51 | 98.26 | 5 | C ARHRRLGAHSPFDY W |
| 2 | CL. 2 | IGHV5-a | 96.52 | 10 | C ATQDYYGSGNYYPDY W |
| 3 | CL. 3 | IGHV5-51 | 99.65 | 1 | C ARFIAVAGPTFDY W |
| 4 | CL. 4 | IGHV5-51 | 98.61 | 4 | C ARGVGGYDSWFDP W |
| 5 | CL. 7 | IGHV5-51 | 99.30 | 2 | C ARRELPYYFDY W |
| 6 | CL. 8 | IGHV5-a | 100 | 0 | C ACSRGY W |
| 7 | CL. 9 | IGHV5-51 | 97.22 | 8 | C ARGTSGSIGY W |
| 8 | CL. 10 | IGHV5-51 | 99.64 | 1 | C ARQEYSSSSGWFDP W |
| 9 | CL. 11 | IGHV5-51 | 100 | 0 | C AVSAGWLQWNWFDP W |
| 10 | CL. 12 | IGHV5-51 | 100 | 0 | C ARHSQRGSHFDY W |
| 11 | CL. 13 | IGHV5-51 | 100 | 0 | C ARHYYDSSGYYYDY W |
| 12 | CL. 15 | IGHV5-a | 96.18 | 11 | C ARHGGAARPDV W |
| 13 | CL. 16 | IGHV5-51 | 99.65 | 1 | C ARIYDSSGYYFDY W |
| 14 | CL. 17 | IGHV5-a | 99.65 | 1 | C AVTLDPKGYYYGMDV W |
| 15 | CL. 18 | IGHV5-51 | 98.26 | 5 | C ARRAYYDSSGYFDY W |
| 16 | CL. 22 | IGHV5-51 | 97.56 | 7 | C ARHSEYSRSAPFGY W |
| 17 | CL. 23 | IGHV5-a | 98.26 | 5 | C TGGTSYDSRDY W |
| 18 | CL. 24 | IGHV5-51 | 98.26 | 5 | C ASRPVYCGGDCYFDY W |

|  | TONS 4  CX_3_CR1+ | IgVH gene | % of IgVH identity | N. of IgVH mutations | CDR3 aminoacid composition |
| --- | --- | --- | --- | --- | --- |
| 1 | CL. 1 | IGHV5-51 | 100 | 0 | C ARLPATMGWFDP W |
| 2 | CL. 2 | IGHV5-51 | 99.30 | 2 | C ARLLFGEMATIRGSGMDV W |
| 3 | CL. 3 | IGHV5-51 | 99.65 | 1 | C ARLGDSFDY W |
| 4 | CL. 4 | IGHV5-51 | 100 | 0 | C ARTMTTVTLDAFDI W |
| 5 | CL. 5 | IGHV5-51 | 100 | 0 | C ARHQDSGSPYYYYYYGMDV W |
| 6 | CL. 6 | IGHV5-a | 100 | 0 | C ARHLSGWYGGDY W |
| 7 | CL. 7 | IGHV5-51 | 100 | 0 | C ARHKGNWGSGYYYYGMDV W |
| 8 | CL. 8 | IGHV5-a | 99.65 | 1 | C ARTHYGSGRIPYYFDN W |
| 9 | CL. 9 | IGHV5-51 | 98.26 | 5 | C ARHRTGYYGSGSYDY W |
| 10 | CL. 10 | IGHV5-a | 100 | 0 | C ARLQAYGGNSL M |
| 11 | CL. 11 | IGHV5-51 | 100 | 0 | C ARHPYYYDSSGYSWYFDY W |
| 12 | CL. 12 | IGHV5-a | 100 | 0 | C ARLDYYDSSGYYGMDV W |
| 13 | CL. 13 | IGHV5-51 | 99.30 | 2 | C ARPNPDYHDSSDYAEGFDP W |
| 14 | CL. 14 | IGHV5-51 | 97.91 | 6 | C ARRHGDYMIDP W |
| 15 | CL. 15 | IGHV5-51 | 100 | 0 | C ARRGDNWFDP W |
| 16 | CL. 16 | IGHV5-51 | 98.26 | 5 | C ARSWGSGSGWFDY W |
| 17 | CL. 17 | IGHV5-51 | 100 | 0 | C ARLPPPPPGAFGSGSYYNSFYFDY W |
| 18 | CL. 18 | IGHV5-51 | 98.61 | 4 | C ARLAVVGTWPSYFDYW G |
| 19 | CL. 19 | IGHV5-a | 100 | 0 | C ARLMGDDYGEYRGYFDY W |
| 20 | CL. 20 | IGHV5-51 | 100 | 0 | C ARSRSAGRGNWFDP W |
| 21 | CL. 21 | IGHV5-51 | 100 | 0 | C ARRVMILGAFDI W |
| 22 | CL. 23 | IGHV5-a | 98.95 | 3 | C ASQEVVTAKDYYYYGMDV W |
| 23 | CL. 24 | IGHV5-51 | 98.61 | 4 | C ARRFVPAAMSIPDDAFDI W |

|  | TONS 4  CX_3_CR1- | IgVH gene | % of IgVH identity | N. of IgVH mutations | CDR3 aminoacid composition |
| --- | --- | --- | --- | --- | --- |
| 1 | CL. 1 | IGHV5-51 | 99.30 | 2 | C ARPQYYYGSGTYGAFDI W |
| 2 | CL. 2 | IGHV5-51 | 99.65 | 1 | C ARSYCGGDCPPWEIDYYGMDV W |
| 3 | CL. 4 | IGHV5-51 | 98.26 | 5 | C ARQQGGDADY W |
| 4 | CL. 5 | IGHV5-51 | 100 | 0 | C ASVVGGTGYFDY W |
| 5 | CL. 6 | IGHV5-51 | 99.65 | 1 | C ARHVLGLSAAGINALNGMDV W |
| 6 | CL. 7 | IGHV5-51 | 99.65 | 1 | C ARTRSSSSYFDP W |
| 7 | CL. 8 | IGHV5-51 | 96.52 | 10 | C ATPRVPG W |
| 8 | CL. 9 | IGHV5-51 | 99.65 | 1 | C AGRTGSSWT W |
| 9 | CL. 10 | IGHV5-51 | 100 | 0 | C ARHKGRDGYKNLNWAIEN W |
| 10 | CL. 11 | IGHV5-51 | 99.65 | 1 | C ARDVLFDY W |
| 11 | CL. 12 | IGHV5-51 | 99.65 | 1 | C ARHISGSSYDSSAYQTAANWFDP W |
| 12 | CL. 13 | IGHV5-51 | 98.95 | 3 | C ARYGVIAEFASLYYFDY W |
| 13 | CL. 14 | IGHV5-a | 99.30 | 2 | C ARRCSSTSCYPSYYYYGMDV W |
| 14 | CL. 15 | IGHV5-51 | 98.26 | 5 | C ATGRDVYSPFDY W |
| 15 | CL. 16 | IGHV5-51 | 98.95 | 3 | C AIEGYCSSTSCPKNYYGMDV W |
| 16 | CL. 17 | IGHV5-a | 100 | 0 | C ARRSGSGTEGYYYYGMDV W |
| 17 | CL. 18 | IGHV5-51 | 98.95 | 3 | C ARSITVAGDFDS W |
| 18 | CL. 19 | IGHV5-51 | 97.56 | 7 | C ARSYSSGWNFDY W |
| 19 | CL. 20 | IGHV5-a | 98.95 | 3 | C ARLTSAGPGV W |
| 20 | CL. 21 | IGHV5-51 | 97.91 | 6 | C ARSGRPQWPYAFDI W |
| 21 | CL. 22 | IGHV5-51 | 97.91 | 6 | C ARGAGIGRPFDY W |
| 22 | CL. 23 | IGHV5-51 | 96.87 | 8 | C ARLREGDSRSWIDY W |

|  | TONS 4  CD27- | IgVH gene | % of IgVH identity | N. of IgVH mutations | CDR3 aminoacid composition |
| --- | --- | --- | --- | --- | --- |
| 1 | CL. 1 | IGHV5-51 | 100 | 0 | C ALSVYYGMDV W |
| 2 | CL. 2 | IGHV5-51 | 98.26 | 5 | C ARLSSGWTGNWFDP W |
| 3 | CL. 3 | IGHV5-51 | 99.30 | 2 | C ARPSGFDYYFDY W |
| 4 | CL. 4 | IGHV5-51 | 99.30 | 2 | C ARSPAFGFGELSSGCIFDY W |
| 5 | CL. 5 | IGHV5-51 | 100 | 0 | C AIRGGDTAMDEIFFDY W |
| 6 | CL. 6 | IGHV5-51 | 100 | 0 | C ARQGPGIAAVYYYGMDV W |
| 7 | CL. 7 | IGHV5-51 | 100 | 0 | C ARLMTNWFDP W |
| 8 | CL. 8 | IGHV5-51 | 100 | 0 | C ARPQRITIFGVVTHGEDDDDY W |
| 9 | CL. 9 | IGHV5-51 | 100 | 0 | C ARLGSSSLGGFGHGYYYYGMDV W |
| 10 | CL. 10 | IGHV5-51 | 100 | 0 | C ASGSGSYPIFDY W |
| 11 | CL. 11 | IGHV5-51 | 100 | 0 | C ARLMGYSGYDPSYYYYGMDV W |
| 12 | CL. 12 | IGHV5-51 | 100 | 0 | C ARRLVLFGELTGWFDP W |

|  | TONS 5  CX_3_CR1+ | IgVH gene | % of IgVH identity | N. of IgVH mutations | CDR3 aminoacid composition |
| --- | --- | --- | --- | --- | --- |
|  |  |  |  |  |  |
| 1 | CL. 1 | IGHV5-a | 100 | 0 | C ARHGEVVRGVIMPRDWFDP W |
| 2 | CL. 2 | IGHV5-51 | 99.30 | 2 | C ARLSYGGNEGCFDY W |
| 3 | CL. 3 | IGHV5-51 | 100 | 0 | C ARATAPYAFDI W |
| 4 | CL. 4 | IGHV5-a | 96.18 | 11 | C ARRRESSNDFDY W |
| 5 | CL. 5 | IGHV5-51 | 100 | 0 | C ARTGIAVAGTDY W |
| 6 | CL. 6 | IGHV5-51 | 98.61 | 4 | C ARVRFLEWFSGFDP W |
| 7 | CL. 7 | IGHV5-51 | 100 | 0 | C ARHDYDSRFDAFDI W |
| 8 | CL. 8 | IGHV5-51 | 100 | 0 | C ARHLAAAPYYYYGMDV W |
| 9 | CL. 9 | IGHV5-51 | 98.26 | 5 | C ARHPYHYDILTGYKYYYGIDV W |
| 10 | CL. 10 | IGHV5-a | 99.30 | 2 | C DLSMVVRGVIRGMDV W |
| 11 | CL. 11 | IGHV51 | 98.95 | 3 | C ARRYYGMDV W |
| 12 | CL. 12 | IGHV5-51 | 100 | 0 | C ARRSSSWGWYFDL W |
| 13 | CL. 13 | IGHV5-51 | 100 | 0 | C ARHVSDLPYGSGSHFDY W |
| 14 | CL. 14 | IGHV5-51 | 100 | 0 | C ARRLEGYYYDSSGY W |
| 15 | CL. 15 | IGHV5-51 | 99.30 | 2 | C ARQNYGASRWYFDL W |
| 16 | CL. 16 | IGHV5-51 | 98.61 | 4 | C ATLPSSSTWLFDY W |
| 17 | CL. 17 | IGHV5-51 | 100 | 0 | C AGGPKYTYGGAYYYYGMDV W |
| 18 | CL. 18 | IGHV5-51 | 99.30 | 2 | C ARQESSGYGSSWSFDY W |
| 19 | CL. 19 | IGHV5-51 | 98.61 | 4 | C ARPTTGIFDC W |
| 20 | CL. 20 | IGHV5-51 | 100 | 0 | C ARLLIQSGSYPAVRYFDL W |
| 21 | CL. 21 | IGHV5-a | 98.95 | 3 | C ARRRESYGEFDY W |
| 22 | CL. 22 | IGHV5-51 | 99.65 | 1 | C ARHWVPDIVVVPAAYDAFDI W |

|  | TONS 5  CX_3_CR1- | IgVH gene | % of IgVH identity | N. of IgVH mutations | CDR3 aminoacid composition |
| --- | --- | --- | --- | --- | --- |
|  |  |  |  |  |  |
| 1 | CL. 1** | IGHV5-51 | 100 | 0 | C ATLFRQQLVQGDAFDI W |
| 2 | CL. 2*** | IGHV5-51 | 100 | 0 | C ARGVRGGSNGYFDY W |
| 3 | CL. 3 | IGHV5-51 | 97.91 | 6 | C ARPTMVRGVMGDYFYYGMDV W |
| 4 | CL. 4 | IGHV5-51 | 99.30 | 2 | C ARVGDSGSYYDDAFDI W |
| 5 | CL. 5* | IGHV5-51 | 96.87 | 9 | C ARRSRGGYCSNISCYVDY W |
| 6 | CL. 6*** | IGHV5-51 | 100 | 0 | C ARGVRGGSNGYFDY W |
| 7 | CL. 7 | IGHV5-51 | 100 | 0 | C ARPGYSSSWYDY W |
| 8 | CL. 8 | IGHV5-51 | 98.95 | 3 | C ARLTVEMATRGMDY W |
| 9 | CL. 9** | IGHV5-51 | 100 | 0 | C ATLFRQQLVQGDAFDI W |
| 10 | CL. 10 | IGHV5-51 | 99.65 | 1 | C ARHPSPSFDYGDNYFDY W |
| 11 | CL. 12 | IGHV5-51 | 100 | 0 | C ASNYYDSSGYPWYFDL W |
| 12 | CL. 13 | IGHV5-51 | 100 | 0 | C ARHGVLTGYYYMDV W |
| 13 | CL. 14 | IGHV5-51 | 99.65 | 1 | C ARGMAAAGGGYFDY W |
| 14 | CL. 15* | IGHV5-51 | 96.87 | 9 | C ARRSRGGYCSNISCYVDY W |
| 15 | CL. 16 | IGHV5-51 | 100 | 0 | C ARHLARGDTHDDMDV W |
| 16 | CL. 17 | IGHV5-51 | 100 | 0 | C ARPHDHSITIFGVVPSDLDV W |
| 17 | CL. 19 | IGHV5-51 | 98.61 | 4 | C ARRGDTSGWSN W |
| 18 | CL. 20 | IGHV5-51 | 98.95 | 3 | C ARPDSGYYSNWYFDL W |
| 19 | CL. 21 | IGHV5-51 | 99.30 | 2 | C ARGVEDYGDYVLFDY W |
| 20 | CL. 22 | IGHV5-51 | 100 | 0 | C ARLRIAVTVADY W |
| 21 | CL. 23 | IGHV5-51 | 98.26 | 5 | C ASPYYGDYDDAFDI W |
| 22 | CL. 24 | IGHV5-51 | 99.65 | 1 | C ARQRYYGSGSPFDY W |

|  | TONS 5  CD27- | IgVH gene | % of IgVH identity | N. of IgVH mutations | CDR3 aminoacid composition |
| --- | --- | --- | --- | --- | --- |
|  |  |  |  |  |  |
| 1 | CL. 1 | IGHV5-51 | 100 | 0 | C ARDWGDYWGQGVTHGENNDDY W |
| 2 | CL. 2 | IGHV5-51 | 100 | 0 | C ARRLVSGYCGSGWFDP W |
| 3 | CL. 3 | IGHV5-51 | 99.65 | 1 | C ARPSGFDYYFDY W |
| 4 | CL. 6 | IGHV5-51 | 100 | 0 | C ARDCSQSWGIGMDV W |
| 5 | CL. 7 | IGHV5-51 | 100 | 0 | C ARLFGVLTQPTHWFDP W |
| 6 | CL. 8 | IGHV5-51 | 99.30 | 2 | C ALRGGGSYDY W |
| 7 | CL. 9 | IGHV5-51 | 100 | 0 | C ARLGSSENRGGHGYYYYGMDV W |
| 8 | CL. 10 | IGHV5-51 | 100 | 0 | C ARRDWGDYWGQG W |
| 9 | CL. 11 | IGHV5-51 | 100 | 0 | C ARGSYSGGGYDPSYYYYGMDV W |
| 10 | CL. 13 | IGHV5-51 | 100 | 0 | C ALSVYYGLDV W |
| 11 | CL. 14 | IGHV5-51 | 100 | 0 | C ARSKDVGATGYCSGGSFDY W |

|  | TONS 6  CX_3_CR1+ | IgVH gene | % of IgVH identity | N. of IgVH mutations | CDR3 aminoacid composition |
| --- | --- | --- | --- | --- | --- |
|  |  |  |  |  |  |
| 1 | CL. 1 | IGHV5-51 | 95.13 | 14 | C ARVHYYDSGTYYKDDS W |
| 2 | CL. 2 | IGHV5-a | 98.95 | 3 | C AWADGAGDITVVTSLGY W |
| 3 | CL. 3 | IGHV5-51 | 98.61 | 4 | C ARPGEAAWYLDL W |
| 4 | CL. 4 | IGHV5-51 | 98.95 | 3 | C ASGDDHGDAFDAFDI W |
| 5 | CL. 5 | IGHV5-a | 98.61 | 4 | C AIDSGWDDAFDI W |
| 6 | CL. 6 | IGHV5-51 | 99.30 | 2 | C ARWFGELLYPDY W |
| 7 | CL. 7 | IGHV5-51 | 95.83 | 12 | C ARHVDTTMGCFDY W |
| 8 | CL. 8 | IGHV5-51 | 99.30 | 2 | C ARLFRGGAPFDY W |
| 9 | CL. 9 | IGHV5-51 | 97.22 | 8 | C ARHATFGVLSNWFDP W |
| 10 | CL. 11 | IGHV5-51 | 97.91 | 6 | C ARQGQRIMSDFDY W |
| 11 | CL. 12 | IGHV5-51 | 99.65 | 1 | C ARHKFPEGIAVAGTWDWFDP W |
| 12 | CL. 14 | IGHV5-51 | 98.61 | 4 | C ARVLKAIVGATGTFDI W |
| 13 | CL. 15 | IGHV5-a | 99.65 | 1 | C ARHSRVNPLRPFVVVPAVTLSLTSFDY W |
| 14 | CL. 16 | IGHV5-a | 99.30 | 2 | C ARLERGDFSPPPYYFDY W |
| 15 | CL. 17 | IGHV5-51 | 100 | 0 | C ARLPPVSGYYAYYAFDI W |
| 16 | CL. 20 | IGHV5-51 | 99.30 | 2 | C ARNSGDGWNDSFDY W |
| 17 | CL. 21 | IGHV5-51 | 100 | 0 | C ARLSADTPMVSPDY W |
| 18 | CL. 22 | IGHV5-a | 100 | 0 | C AGGLPQRDWSYNYYGMDV W |
| 19 | CL. 23 | IGHV5-51 | 98.61 | 4 | C ARLLRFGELYPFDY W |
| 20 | CL. 24 | IGHV5-51 | 98.26 | 5 | C ARRNGDYAGGFYYYGMDV W |

|  | TONS 6  CX_3_CR1- | IgVH gene | % of IgVH identity | N. of IgVH mutations | CDR3 aminoacid composition |
| --- | --- | --- | --- | --- | --- |
|  |  |  |  |  |  |
| 1 | CL. 2** | IGHV5-51 | 100 | 0 | C ARFWHYFDY W |
| 2 | CL. 4 | IGHV5-51 | 100 | 0 | C ARQHIAAAGFAYFDY W |
| 3 | CL. 6 | IGHV5-a | 99.30 | 2 | C ARQNLHYGDAFDI W |
| 4 | CL. 7* | IGHV5-a | 96.18 | 11 | C ARHEDSMRAFDY W |
| 5 | CL. 8 | IGHV5-51 | 97.91 | 6 | C ARRLTYGGEINWFDS W |
| 6 | CL. 9** | IGHV5-51 | 100 | 0 | C ARFWHYFDY W |
| 7 | CL. 10 | IGHV5-51 | 98.26 | 5 | C ARPRGRRDGYNGRAFDI W |
| 8 | CL. 11 | IGHV5-51 | 97.22 | 8 | C ARQEFTYGGFDY W |
| 9 | CL. 12 | IGHV5-51 | 98.26 | 5 | C AIRSCSSTSCGFDY W |
| 10 | CL. 13* | IGHV5-a | 96.18 | 11 | C ARHEDSMRAFDY W |
| 11 | CL. 14 | IGHV5-a | 99.30 | 2 | C ARRRMYSGNSWSHDY W |
| 12 | CL. 16 | IGHV5-51 | 98.26 | 5 | C ARQVSRYYFDY W |
| 13 | CL. 17 | IGHV5-51 | 98.61 | 4 | C ARLTYYSRYFDY W |
| 14 | CL. 18 | IGHV5-51 | 99.65 | 1 | C ARLYYDILTRWAAPFDY W |
| 15 | CL. 19 | IGHV5-51 | 99.30 | 2 | C ARRGDFGDYADY W |
| 16 | CL. 20 | IGHV5-51 | 97.22 | 8 | C ARLMWHFAVSAFDY W |
| 17 | CL. 22 | IGHV5-51 | 99.65 | 1 | C ARPARENYHYYYMDV W |
| 18 | CL. 23 | IGHV5-a | 100 | 0 | C ARQEAFYFASGTYYSGLDV W |
| 19 | CL. 24 | IGHV5-a | 98.95 | 3 | C ARHSQDIVVVPAGFDY W |

|  | TONS 6  CD27- | IgVH gene | | % of IgVH identity | N. of IgVH mutations | CDR3 aminoacid composition |
| --- | --- | --- | --- | --- | --- | --- |
|  |  |  | |  |  |  |
| 1 | CL. 1 | IGHV5-51 | | 100 | 0 | C ASSYGAFEYGMDV W |
| 2 | CL. 2 | IGHV5-51 | | 100 | 0 | C ARLAGYSLYYYYGMDV W |
| 3 | CL. 3 | IGHV5-51 | | 100 | 0 | C ARPHGGSDYKVDY W |
| 4 | CL. 4 | | IGHV5-a | 100 | 0 | C ARLGGGFGELHQNYYGMDV W |
| 5 | CL. 5 | | IGHV5-a | 100 | 0 | C ASGRESSSWYDY W |
| 6 | CL. 6 | IGHV5-51 | | 99.65 | 1 | C ARPVDDILTGYNLDAFDI W |
| 7 | CL. 7 | | IGHV5-a | 100 | 0 | C ARRQYGPDY W |
| 8 | CL. 9 | | IGHV5-a | 99.30 | 2 | C ARQYYYDISGFEGNWFDP W |
| 9 | CL. 10 | IGHV5-51 | | 100 | 0 | C ARLDCSSTRCYFNHYFHYYGMDV W |
| 10 | CL. 11 | | IGHV5-a | 100 | 0 | C ASQASYVWGSYRWHAFDI W |
| 11 | CL. 12 | IGHV5-51 | | 100 | 0 | C ARTTQRWLQSQAAWTSFHY W |

**Supplemental Table 1 online.** Antigen selection, as determined by significant accumulation of R mutations in the complementary determining region (CDR) regions and/or significant preservation of framework (FR) amino acid sequences, was observed in similar proportions of mutated V_H_ sequences both in the CX_3_CR1^+^ and CX_3_CR1^-^ GC B cell subsets (8/35 and 4/19 clones respectively. Asterisks indicate clonal relatedness.
